# Encephalocele: A Case Series From Abuja, North Central Nigeria

**DOI:** 10.7759/cureus.23249

**Published:** 2022-03-17

**Authors:** Ugo Ugwuanyi, Obinna Ayogu, Daniel E Onobun, Morayo Salawu, Chizimenu O Mordi

**Affiliations:** 1 Neurosurgery, National Hospital Abuja, Abuja, NGA; 2 Neurosurgery, Wellington Neurosurgery Centre, Abuja, NGA; 3 Neurological Surgery, Wellington Neurosurgery Centre, Abuja, NGA; 4 Anesthesiology, National Hospital Abuja, Abuja, NGA; 5 Neurology, Wellington Neurosurgery Center, Abuja, NGA

**Keywords:** outcome, vp shunt, repair, hydrocephalus, encephalocele

## Abstract

An encephalocele is a congenital tube defect in which there is an extension of intracranial structures outside the normal confines of the skull. Its presentation at birth provokes a lot of anxiety amongst parents, guardians and care providers with regards to compatibility with life, surgical treatments and effects on developmental milestones and higher mental functions. This report is on our initial experience in the first six months following surgical treatment of four consecutive cases presenting in infancy. The aim of this case series is to report our initial experience of the management of encephaloceles using four consecutive cases that presented in infancy. A review of four infants who presented to our neurosurgery service was conducted including patterns of presentation, neuroimaging findings, scope of surgical intervention and neurological outcome at the six-month review. The results are presented in short case reports and summarized in a table. Two five-month-old females, one six-month-old female and one two-month-old female infants presented to our neurosurgery clinic with progressively increasing encephaloceles at different locations. Brain MRI revealed meningo-encephalocele in all, but with associated hydrocephalus in two cases only. They all had excision and repair of encephalocele under the same general anaesthesia while only two had a ventriculoperitoneal (VP) shunt. Developmental milestones were on course at 6 months follow-up following discharge. Although the presentation of encephaloceles can be frightening to parents and care providers, careful clinical and radiological evaluation is a recipe for sound surgical planning and improved outcome.

## Introduction

Encephalocele is a congenital neural tube anomaly that is characterized by an extension of the normal intracranial structures outside the normal confines of the skull [1]. It is seen in 1 per 5000 live births globally, and about 70% of encephaloceles are occipital [2]. Occipital encephalocele often presents as swelling that overlies the occipital bone and is often more common in females than males [3]. This can present as a giant occipital encephalocele in which the size of the swelling is bigger than the size of the patient’s head. Fronto-ethmoidal encephaloceles constitute about 15% of all encephaloceles and involve encephaloceles in the following locations: naso-ethmoidal, naso-frontal and naso-orbital [1,4]. The outcome of the management depends on the time of presentation, its size, content and associated complications such as hydrocephalus. Treatment can be challenging if the patient presents late and will require a more detailed evaluation and meticulous surgical repair. Cranio-facial disfigurement is often a major source of anxiety and is quite frightening to the parents and care providers. Several misinterpretations have been given to these cases, consequently, most have been wasted as monsters and evil children that portend a bad omen for the family. Others were taken to traditional medicine homes for ritual cleansing of imaginary evil spirits and witchcraft. But currently, an increasing number spend some time in prayer houses before eventually presenting late to the hospital. This report aims to outline the clinical strategies deployed in managing a few cases who made it to the hospital, treatments given and the eventual outcome which should hopefully provide a platform to encourage advocacy towards early presentation to specialized centres in order to save more of these infants from neglect, and delayed and untoward treatments. Following the necessary institutional ethical approvals and consent from the parents and care providers of these infants, a retrospective review of four infants who presented to our neurosurgery service was conducted including patterns of presentation, neuroimaging findings, scope of surgical intervention and neurological outcome in six months review. Results are presented in concise case reports and also summarized in a table.

## Case presentation

Case 1

**Figure 1:**
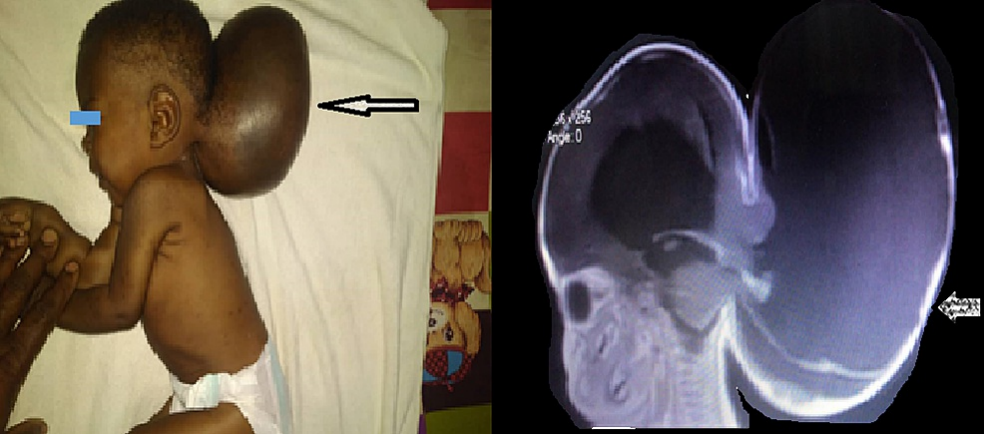
Pre-op evaluation of suboccipital cystic, transilluminating swelling with MRI; herniation of occipital and cerebellar lobes into a sub-occipital encephalocele with associated hydrocephalus.

**Figure 2:**
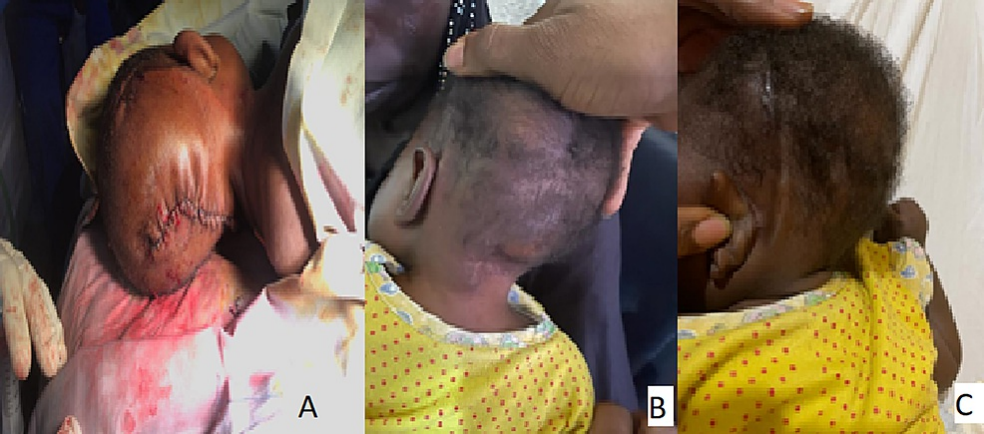
A - Immediate post-op (VP shunt and repair of sub-occipital encephalocele); B - One month post discharge; C - Six months follow-up. VP: ventriculoperitoneal

A five-month-old female child presented with a five-month history of progressive occipital swelling that was noticed from birth. Her mother did not attend any antenatal service during pregnancy and was not on any folic acid before conception. Clinical examination showed a stable and playful child with a large fluctuant, transilluminating occipital swelling almost the size of her head. Occipitofrontal circumference (OFC)was 58 cm with bulging and tense anterior fontanelle. Brain MRI scan showed features in keeping with a large meningo-encephalocele with hydrocephalus (Figure 1). She had ventriculoperitoneal (VP) shunt insertion, surgical excision and repair of occipital encephalocele. At six months follow-up, developmental milestones were on schedule (Figure 2).

Case 2

**Figure 3:**
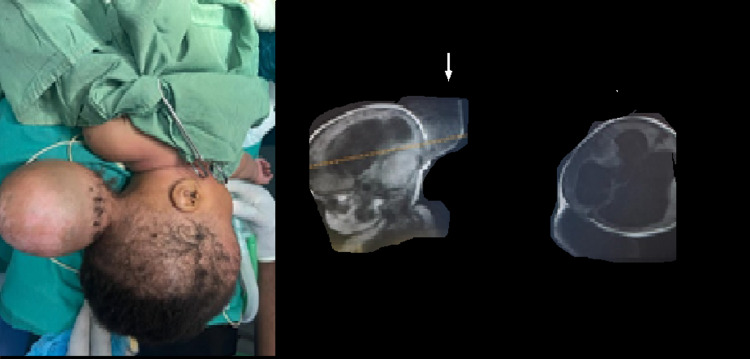
Pre-op evaluation of suboccipital cystic, transilluminating swelling with MRI Brain showing herniation of brain tissue and CSF into the suboccipital encephalocele CSF: cerebrospinal fluid

**Figure 4:**
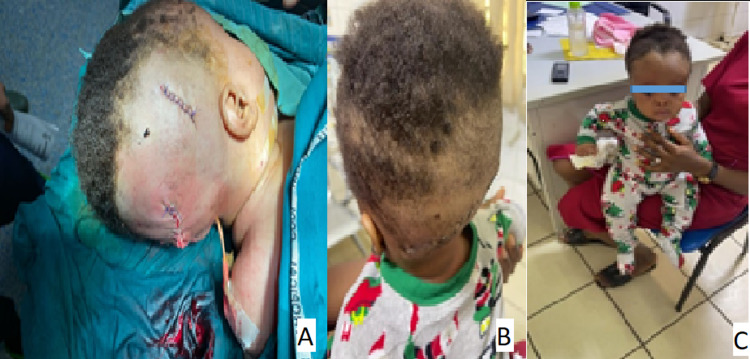
A - Immediate post-op scar for VP shunt and repair of sub-occipital encephalocele; B - One month follow-up; C - Six months follow-up. VP: ventriculoperitoneal

A five-month-old female infant presented with swelling at the back of the head, however, her mother attended an antenatal clinic and was on her routine antenatal medications. Clinical examination showed a stable infant, with marked craniofacial disproportion, tense anterior fontanelle and OFC of 57 cm. She had a huge fluctuant non-tender occipital, transilluminating mass, measuring 15 cm by 8 cm. Brain MRI done showed features in keeping with occipital meningo-encephalocele and hydrocephalus (Figure 3). She had VP shunt insertion with excision and repair of occipital encephalocele. She was discharged to outpatient follow-up, and at six months review, her milestones were satisfactory (Figure 4).

Case 3

**Figure 5:**
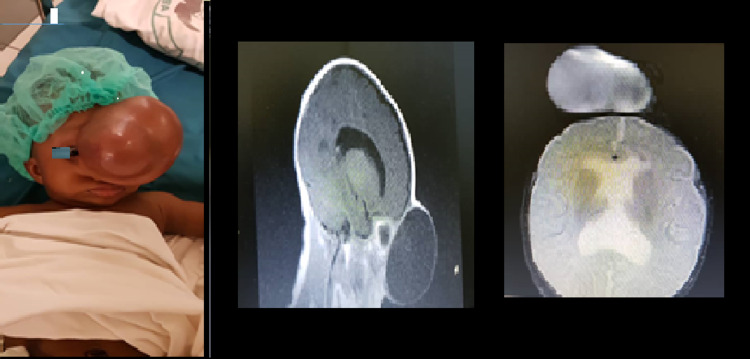
Pre-op evaluation of frontonasal cystic, trans-illuminating mass and MRI showing mostly a cystic frontonasal mass with no brain tissue herniation but with associated hydrocephalus.

**Figure 6:**
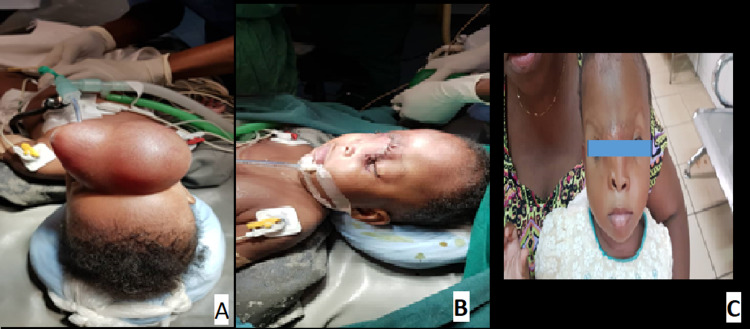
A - Intra-op image; B - Immediate post-op image showing nasal bridge scar; C - six months follow-up.

A six-month-old female presented with progressively increasing mass at the nasal bridge, occluding the eyes and nose with increasing difficulty in breathing. Mother had full antenatal clinic** **and was on folic acid. Clinical examination showed a multilobed non-tender cystic mass originating from the nasal bridge and overlying the face. The OFC was 45 cm. Brain MRI showed features in keeping with frontoethmoidal meningo-encephalocele (Figure 5). She had surgical excision and repair of frontoethmoidal encephalocele with reasonably good outcome and milestones at six months (Figure 6). 

Case 4

**Figure 7:**
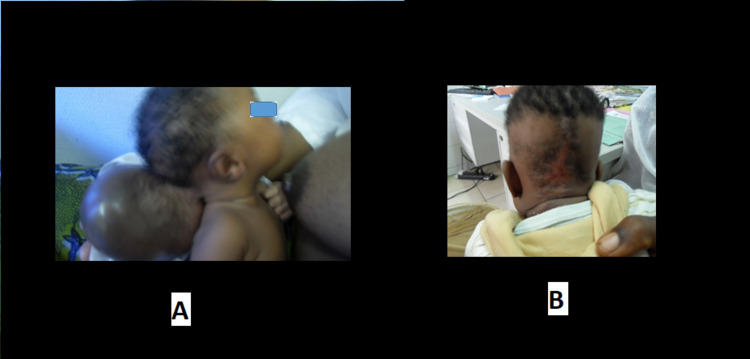
A - Sub-occipital encephalocele pre-operatively; B - Healed scar at six months follow-up.

A two-month-old female infant was rescued from an internally displaced persons (IDP) camp with swelling at the back of the head. The mother did not attend any antennal clinic for obvious reasons. Clinical examination showed a stable infant, with a normal OFC of 37 cm. She had a huge fluctuant non-tender occipital, transilluminating mass, measuring 15 cm by 8 cm. Brain MRI done showed features in keeping with occipital meningo-encephalocele but no hydrocephalus. She had a repair of the occipital encephalocele and suffered wound infection and breakdown which increased hospital stay to 4 weeks but subsequently discharged to outpatient follow-up. At six months review, milestones were satisfactory (Figure 7).

Table 1 provides a summary of the cases.

**Table 1:** Summary of the cases VP: ventriculoperitoneal; OFC: occipitofrontal circumference; ANC: antenatal clinic

SN	Age months	Sex	Presentation	ANC	Folate	OFC (cm)	MRI	Suwanwela class	VP shunt	Repair	Milestones at 6 months
1	5	F	Swelling back of head	No	No	58	Meningo-encephalocele Hydrocephalus	suboccipital	Yes	Yes	On schedule
2	5	F	Swelling back of head	Yes	Yes	57	Meningo-encephalocele Hydrocephalus	suboccipital	Yes	Yes	On schedule
3	6	F	Frontonasal swelling Difficulty suckling and swallowing	Yes	Yes	46	Meningo-encephalocele	Fronto-nasoethmoidal	No	Yes	On schedule
4	2	F	Swelling back of head	No	No	37	Meningo-encephalocele	suboccipital	No	Yes	On schedule

## Discussion

Encephalocele commonly is a congenital anomaly but can be acquired following intracranial space-occupying lesions like brain tumours [5]. Based on the nature of its content, it is usually divided into four categories, which include: meningoceles (herniation of the meninges and the cerebrospinal fluid [CSF]), meningoencephaloceles (contains the meninges, brain tissue and CSF), glioceles (glial lined cyst with CSF) and atretic cephaloceles (dura, fibrous tissue and atretic brain tissue) [6]. All cases studied presented with fluctuant, soft swelling on the head and it is clear from MRI findings that the meningoencephalocele variety was the commonest seen thus far in our environment. Another classification system (Suwanwela and Suwanwela) uses the location of the encephalocele and classifies it into occipital, fronto-ethmoidal, cranial vault, basal and posterior fossa encephalocele [7]. Again, from the series presented it is obvious that the occipital/suboccipital type was the commonest constituting three out of the four cases while the frontonasal-ethmoidal presented only one case. Reasons for this is not known but probably due to the delayed closure of the cranial neuropore at the suboccipital/occipital region.

Both genetic and environmental factors have been suggested in the etiology, however, details of its cause remain poorly understood. This condition is seen more in children born in families with low socioeconomic status, and maternal folate deficiency has also been implicated, but there is still a paucity of data to confirm [8]. From the above series, up to half of the cases had adequate exposure to antenatal care and folic acid use during pregnancy. It is however not clear if such use was started early enough to cover the period of embryogenesis. In the other two cases, poverty was profound, and in one case, the infant was rescued from an internally displaced persons (IDP) camp. It was therefore easy to extrapolate that due to malnutrition, inadequate exposure to folic acid supplements at the appropriate time during embryogenesis was a major factor in causation.

Radiologic evaluation of these patients includes CT and MRI, which characterized the lesion and its contents as well as the anatomy of the bony defect. But perhaps, more important for surgical planning and safety is angiography studies for vascular involvement [9]. This is because most of the encephaloceles are midline structures, therefore relationships with the midline venous sinus is a surgically useful information.

The choice and extent of surgery are determined by pre-operative clinical and radiological findings. For the two cases with encephalocele associated with hydrocephalus, the surgical principle adopted was to first construct a VP shunt to divert the CSF from the encephalocele. Then followed on the same general anaesthesia with muscle relaxation by the excision and repair of the hernial defect. Following the excision of redundant brain tissue which is believed to be non-viable, the overlying dura is repaired in a watertight fashion with a continuous 3.0 vicryl suture. Then the galea layer and finally the skin is repaired with 3.0 nylon after excising the redundant skin to achieve a reasonably acceptable cosmetic look. Diversion of the CSF flow from the repair prevents fistula and wound breakdown. The other two cases that did not have associated hydrocephalus had a straightforward repair of encephalocele under general anaesthesia as outlined above. Wound healing was perfected in 10 days post-op and three cases were discharged within two weeks. Unfortunately, one of the cases stayed extra two weeks following suboccipital wound breakdown due to infection and undernutrition.

It appeared that all the four cases studied were on schedule with developmental milestones at 6 months follow-up. They had all achieved psychomotor milestones appropriate for age, however, further follow-up over the years will determine appropriate higher mental functions development. The probable determinants of outcome perhaps have been documented to include the volume and eloquence of herniated brain tissue content as well as the size of the encephalocele. Larger size and increased volume of herniated brain tissue confer a worse prognosis [10]. It is for the reasons of eloquence that the fronthoethmoidal ones are believed to have a better outcome even though less than 5% of infants with encephalocele develop normally [11].

## Conclusions

Although the presentation of encephaloceles can be frightening to parents and care providers, careful clinical and radiological evaluation is a recipe for sound surgical planning and improved outcome. In our case series, the initial six-month follow-up revealed acceptable psychomotor development, although a longer term follow-up is required to achieve better determination of higher mental function development. In the meantime, parents and care providers have a lot to savour in terms of the magical cosmetic transformation and quality of life for these cases. There should therefore be an intensified effort towards sensitization and advocacy towards early presentation of these cases.
